# CatWalk XT gait parameters: a review of reported parameters in pre-clinical studies of multiple central nervous system and peripheral nervous system disease models

**DOI:** 10.3389/fnbeh.2023.1147784

**Published:** 2023-06-07

**Authors:** Ivanna K. Timotius, Reinko F. Roelofs, Bar Richmond-Hacham, Lucas P. J. J. Noldus, Stephan von Hörsten, Lior Bikovski

**Affiliations:** ^1^Department of Electronics Engineering, Satya Wacana Christian University, Salatiga, Indonesia; ^2^Department of Experimental Therapy, University Hospital Erlangen and Preclinical Experimental Animal Center, Friedrich-Alexander-University Erlangen-Nürnberg, Erlangen, Germany; ^3^Noldus Information Technology BV, Wageningen, Netherlands; ^4^Myers Neuro-Behavioral Core Facility, Sackler Faculty of Medicine, Tel Aviv University, Tel Aviv-Yafo, Israel; ^5^Donders Center for Neuroscience, Radboud University, Nijmegen, Netherlands; ^6^School of Behavioral Sciences, Netanya Academic College, Netanya, Israel

**Keywords:** CatWalk XT, gait, rodents, mouse, CNS, PNS, locomotor deficits

## Abstract

Automated gait assessment tests are used in studies of disorders characterized by gait impairment. CatWalk XT is one of the first commercially available automated systems for analyzing the gait of rodents and is currently the most used system in peer-reviewed publications. This automated gait analysis system can generate a large number of gait parameters. However, this creates a new challenge in selecting relevant parameters that describe the changes within a particular disease model. Here, for the first time, we performed a multi-disorder review on published CatWalk XT data. We identify commonly reported CatWalk XT gait parameters derived from 91 peer-reviewed experimental studies in mice, covering six disorders of the central nervous system (CNS) and peripheral nervous system (PNS). The disorders modeled in mice were traumatic brain injury (TBI), stroke, sciatic nerve injury (SNI), spinal cord injury (SCI), Parkinson’s disease (PD), and ataxia. Our review consisted of parameter selection, clustering, categorization, statistical evaluation, and data visualization. It suggests that certain gait parameters serve as potential indicators of gait dysfunction across multiple disease models, while others are specific to particular models. The findings also suggest that the more site-specific the injury is, the fewer parameters are reported to characterize its gait abnormalities. This study strives to present a clearly organized picture of gait parameters used in each one of the different mouse models, potentially helping novel CatWalk XT users to apply this information to similar or related mouse models they are working on.

## Graphical Abstract

The circular bar plots depict that publications of a system injury reported more CatWalk gait parameters in comparison to publications of a site-specific injury. 
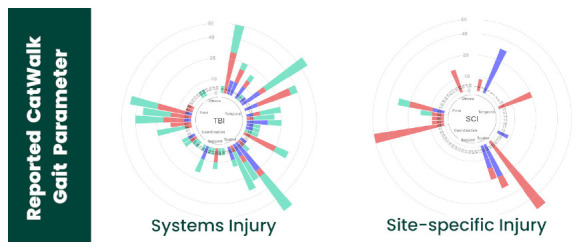

## 1. Introduction

Adaptation of gait dynamics is a prominent symptom of many neurological and movement disorders and injuries, such as sciatic nerve injury (Bozkurt et al., 2008), spinal cord injury (Hendriks et al., 2006), traumatic brain injury (Neumann et al., 2009), multiple sclerosis (Bernardes et al., 2017), Parkinson’s disease, Huntington disease, and stroke (Vandeputte et al., 2010). Over the last few decades, various methods have been developed for the assessment of rodent gait dynamics. Among the earliest methods for evaluating gait parameters are static footprint analysis (i.e., ink-based foot analysis), open-field observations, the Basso, Beattie, and Bresnahan (BBB) score (Basso et al., 1995), and Basso Mouse Scale (BMS) score (Basso et al., 2006).

**FIGURE 1:**
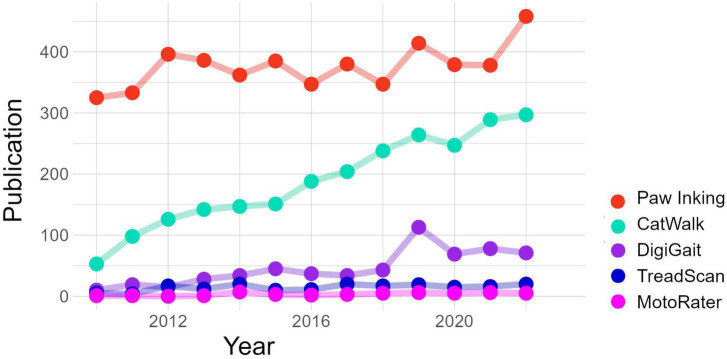
The number of publications per year of studies using commercial gait analysis systems, compared to the most used non-commercial method, as collected using Google Scholar. CatWalk XT (Noldus Information Technology, BV), DigiGait™ (Mouse Specifics, Inc.), TreadScan (Clever Sys Inc.), and MotoRater (TSE Systems GmbH).

Currently, many researchers use automated behavioral tests to observe and quantify the gait performance of rodents. Automated gait analysis methods provide objective, reliable, and sensitive measurements of locomotor and gait abnormalities compared to earlier methods (Garrick et al., 2021). Moreover, the automation of gait analysis has enabled the monitoring of a new set of time-based parameters that were previously impossible to observe, such as swing duration and inter-limb coordination (Kappos et al., 2017). Finally, automation has facilitated the assessment of mouse gait and made it possible for more users to perform gait assessments in mice, resulting in an increased number of published studies and increased variety of tested models (Figure 1).

CatWalk XT, developed by Noldus Information Technology, was among the first commercially available automated system for gait analysis. CatWalk XT is a camera-based system that quantifies gait performance in rodents. The use of CatWalk XT in various rodent models of human neurological diseases has steadily increased since its initial development by Hamers et al. (2001, 2006). CatWalk XT has been instrumental in increasing the popularity of gait analysis as a valuable behavioral endpoint, making it one of the most used automated gait analysis systems worldwide (Figure 1).

As an automated gait analysis system, CatWalk XT has the advantage of generating a large number of gait parameters easily and reliably in freely moving mice, in contrast to treadmill-based systems where animals are forced to walk. Moreover, unlike treadmill systems, CatWalk XT makes use of complete internal reflection of green light within a glass walkway. The green light escapes the glass walkway when the paw of an animal that traverses the walkway touches the glass, and it is reflected by the tissue of the paw toward a 100 frame per second color camera positioned below the glass walkway. Due to this principle, only actual footfalls are detected, and the beginning and end of each footfall can be determined with 10 ms accuracy (i.e., 1 frame). This highly precise spatial and temporal detection of footfalls allows the CatWalk XT software to generate over 200 static and dynamic gait parameters for rats and mice (Timotius et al., 2018a, 2019).

The CatWalk XT system can identify impairments in multiple gait and motor function parameters, including Stride Length, Stand Time, Swing Time, Duty Cycle, Regularity Index, Print Area, and Swing Speed (see Table 1 for additional parameters). Moreover, the system is able to identify subtle changes in paw use and movement patterns that may have gone undetected with non-automated assays of freely moving animals. The comprehensive set of gait parameters provided by CatWalk XT allows researchers to gain an in-depth understanding of how different forms of central nervous system (CNS; e.g., ataxia, Parkinson’s disease, traumatic brain injury, stroke, and spinal cord injury) and peripheral nervous system (PNS; e.g., sciatic nerve injury) injuries affect motor and gait function. A recent paper demonstrated the effectiveness of the CatWalk XT system in an experimental stroke model by revealing significant alterations in 21% of the analyzed CatWalk XT gait parameters (Chen et al., 2020). One study of a Parkinson’s disease (PD) mouse model evaluated 62 CatWalk XT gait parameters and indicated 15 to be more closely related to the animal’s genotype (Radlicka et al., 2023). Another study detected differences in 84 CatWalk XT gait parameters between a specific PD mouse model and its wild-type littermates (Timotius et al., 2018a, 2019). In a study of an ataxia mouse model (Perez et al., 2021), mice with ataxia experienced a progressive deterioration in their motor coordination, as evidenced by their decreased use of three paws (as opposed to one, two, or four paws) while crossing the CatWalk XT walkway. Additionally, mice displayed slower speed and cadence during each run across the platform, possibly indicative of muscle weakness. In the sciatic nerve injury (SNI) model, it was reported that the coordination-related CatWalk XT gait parameters were highly correlated with neuropathic pain severity and response to analgesic treatment (Xu et al., 2019). In an experimental thoracic injury model, Zheng et al. (2023) assessed paw intensity and size; however, as these are parameters affected by weight, they developed a ratio score (hind-limb/fore-limb ratio of pixel intensity of paw prints and paw print size). They discovered that such a ratio was highly effective in evaluating locomotor function impairment and recovery (Zheng et al., 2023).

**TABLE 1:** The CatWalk XT parameters and grouping of parameters used in Figure 2.

No.	CatWalk XT parameters	Group
1	Run duration/other statistics duration	Temporal
2	Run average speed/other statistics average speed/body speed mean	Temporal
3	Run maximum variation/other statistics maximum variation/body speed variation mean	Temporal
4	Stand (s) mean	Temporal
5	Stand (s) SD*	Temporal
6	Stand index mean	Temporal
7	Max contact at (%) mean	Temporal
8	The mean of max intensity at (%)	Temporal
9	Swing (s) mean	Temporal
10	Swing (s) SD*	Temporal
11	Swing speed (cm/s) mean	Temporal
12	Swing speed (cm/s) SD*	Temporal
13	Step cycle (s) mean	Temporal
14	Duty cycle (%) mean	Temporal
15	Single stance (s) mean	Temporal
16	Initial dual stance (s) mean	Temporal
17	Terminal dual stance (s) mean	Temporal
18	Other statistics number of steps	Temporal
19	Other statistics cadence	Temporal
20	Right hip/right knee/left hip/left knee/nose/abdomen/tail/genitalia (%)	Temporal
21	Phase dispersion parameters/phase lag	Temporal
22	Coupling parameters	Temporal
23	Stride length (cm) mean or normalized stride length	Spatial
24	BOS front paws mean (cm)	Spatial
25	BOS hind paws mean (cm)	Spatial
26	Print positions	Spatial
27	Sway parameters	Spatial
28	Support zero (%)	Support
29	Support single (%)	Support
30	Support diagonal (%)	Support
31	Support girdle (%)	Support
32	Support lateral (%)	Support
33	Support three (%)	Support
34	Support four (%)	Support
35	Step sequence number of patterns	Coordination
36	Step sequence CA (%)*	Coordination
37	Step sequence CB (%)*	Coordination
38	Step sequence AA (%)*	Coordination
39	Step sequence AB (%)*	Coordination
40	Step sequence RA (%)*	Coordination
41	Step sequence RB (%)*	Coordination
42	Step sequence regularity index (%)	Coordination
43	Max contact area (cm^2^) mean	Print
44	Print length (cm) mean	Print
45	Print width (cm) mean	Print
46	Print area (cm^2^) mean	Print
47	Toe spread (cm) mean	Print
48	Paw angle body axis (°) mean	Print
49	Paw angle movement vector (°) mean	Print
50	Intermediate toe spread	Print
51	Sciatic functional index (SFI)	Print
52	Fibular functional index (FFI)	Print
53	Intensity ratio and asymmetry	Others
54	Difference score	Others
55	Combined CatWalk index (CCI)	Others
56	Stand ratio/stride length ratio/step cycle ratio/swing speed ratio/print length ratio/print width ratio	Others
57	Phase dispersion asymmetry/coupling asymmetry	Others
58	Print area RH/LH* ratio or duty cycle RH/LH* ratio	Others

*RF, right fore; LF, left fore; RH, right hind; LH, left hind; SD, standard deviation; CA, cruciate sequence (RF-LF-RH-LH); CB, cruciate sequence (LF-RF-LH-RH); AA, alternate sequence (RF-RH-LF-LH); AB, alternate sequence (LF-RH-RF-LH); RA, rotary sequence (RF-LF-LH-RH); RB, rotary sequence (LF-RF-RH-LH).

One limitation of automated systems is that extracting a wide array of parameters can pose a challenge, namely selecting the most appropriate data and/or parameters that define the changes within the disease model being examined. One method for parameter selection is to rely on previous publications. However, this approach may not always be viable since not all studies report the same parameters, and replication may not be possible (Timotius et al., 2018a, 2019). Nevertheless, by accessing data from multiple studies and identifying key significant parameters replicated for each animal model, we can provide a review of these parameters that (1) may indicate differences between focal and non-focal insult models (e.g., SNI vs. stroke); (2) may serve as main alerting parameters of general gait dysfunction, as these would be affected irrespective of the mouse model; or (3) may help differentiate between mouse models, enabling to use gait analysis for differential diagnosis between various biological and/or neurological insults.

## 2. Materials and methods

**FIGURE 2:**
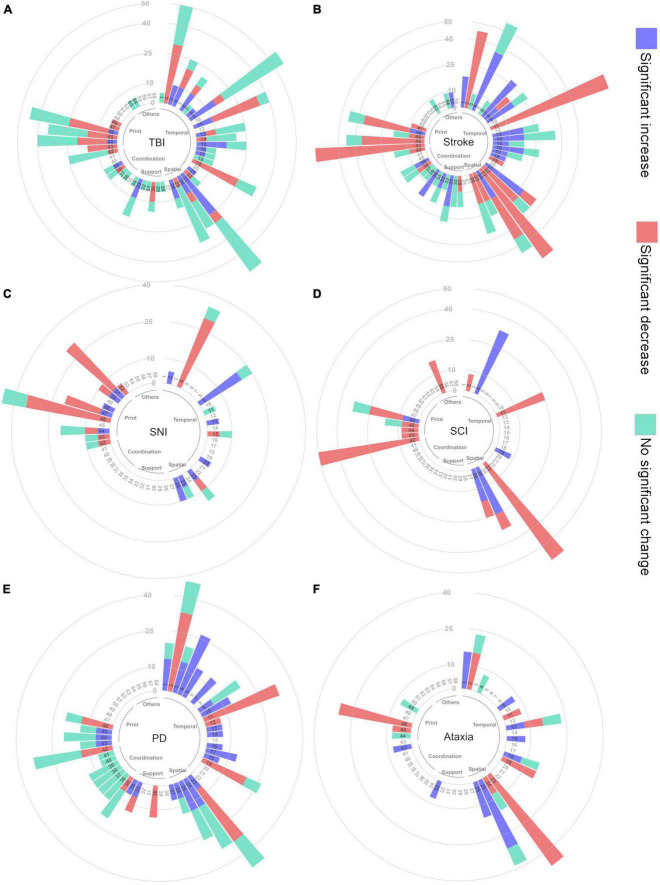
Circular bar plot depicting the percentage of publications reporting specific CatWalk XT gait parameters **(A)** TBI, **(B)** stroke, **(C)** SNI, **(D)** SCI, **(E)** PD, **(F)** ataxia. The names of the corresponding gait parameters are listed in Table 1.

We collected and examined a total of 91 published pre-clinical studies using the CatWalk XT analysis system related to traumatic brain injury (TBI), stroke, sciatic nerve injury (SNI), spinal cord injury (SCI), Parkinson’s disease (PD), and ataxia. The electronic search was performed using several search engines (i.e., PubMed and Google Scholar) and ended in March 2022. The keywords used in the search process were CatWalk, rodents, mouse, mice, and the diseases of interest for our study. The majority of the studies (86%) were published between 2017 and 2022 (Baiguera et al., 2012; Bernardes et al., 2017; Caballero-Garrido et al., 2017; Carboni et al., 2017; Chen et al., 2017, 2019, 2020; Cline et al., 2017; Fujimaki et al., 2017; Hayashi et al., 2017; Hsieh et al., 2017; Matyas et al., 2017; Mountney et al., 2017; Ni et al., 2017; Pöttker et al., 2017; Rocca et al., 2017; Roth et al., 2017; Schönfeld et al., 2017; Crowley et al., 2018, 2019; Du et al., 2018a,b; Frahm et al., 2018; Fröhlich et al., 2018; Liu et al., 2018, 2019; Lu et al., 2018; Mozafari et al., 2018; Noristani et al., 2018; Okuwa et al., 2018; Sowa et al., 2018; Su et al., 2018; Timotius et al., 2018b,^2021^; Truong et al., 2018; Zhu et al., 2018; Cross et al., 2019; Guy et al., 2019; Kolosowska et al., 2019; Leite et al., 2019; Ma et al., 2019; Minakaki et al., 2019; Minarelli et al., 2019; Miterko et al., 2019; Pacheco et al., 2019; Thau-Zuchman et al., 2019; Bengoa-Vergniory et al., 2020; Campos-Pires et al., 2020; Chung et al., 2020; Goldshmit et al., 2020; Goncalves et al., 2020; Heinzel et al., 2020a; Henry et al., 2020; Lin et al., 2020, 2022; Murphy et al., 2020; Namdar et al., 2020; Neckel et al., 2020; Pinkowski et al., 2020; Ritzel et al., 2020, 2021; Schweizer et al., 2020; Walter et al., 2020; Weber et al., 2020; Wilke et al., 2020; Bärmann et al., 2021; Deng et al., 2021; Gries et al., 2021; Huang et al., 2021, 2022; Knorr et al., 2021; Mallah et al., 2021; Niewiadomska-Cimicka et al., 2021; Perez et al., 2021; Wang et al., 2021; Zheng et al., 2021; Chai et al., 2022; Haas et al., 2022; Wertheim et al., 2022). The rest of the studies were published in 2016 or before (Baiguera et al., 2012; Hetze et al., 2012; Wang et al., 2012; Balkaya et al., 2013; Streijger et al., 2013; Casadei et al., 2014; Forgione et al., 2014; Qin et al., 2014; Rotermund et al., 2014; Tsika et al., 2014; Hayakawa et al., 2015; Saal et al., 2015; Kyriakou et al., 2016; Tatenhorst et al., 2016; Vidal et al., 2017). The details of the publications are summarized in Table 2. An important element in comparing studies in research synthesis or systematic review is determining publication bias and data heterogeneity within and across studies. To minimize the potential for bias, we implemented several steps to ensure the rigor and quality of the included articles. These steps included retrieving papers with no conflicts of interest, examining the methods and results and ensuring that sufficient numbers of animals were used per group (*n* ≥ 10, with exceptions of two publications in PD and one publication in SCI, where *n* ≤ 10, which were included due to the limited number of publications using mouse models), and sourcing all papers from indexed journals that employ a rigorous review process (see more information on journal name and impact factor in Supplementary material). From the collected publications, we examined which gait parameters were reported. For each gait parameter used, we evaluated whether any significant difference was reported in the publications compared to healthy controls or baseline levels. The numbers of publications in percent reported for all gait parameters are presented in a circular stack bar plot (Figure 2). In the event that, due to certain circumstances, one independent study reported both a significant increase and a significant decrease in a given gait parameter, the circular stack bar plot displays the differences that were more prominent or prevalent. The gait parameters were arranged in the circular stack bar plot according to their main category. Currently, there is no universal gait parameter grouping; rather, the grouping depends on the experimental model used and the research question of interest. In this study, inspired by Caballero-Garrido et al. (2017), we have grouped the gait parameters into six categories: temporal, spatial, support, coordination, print, and others. The grouping of these gait parameters is listed in Table 1.

**TABLE 2:** Experimental models and the number of publications that utilized CatWalk XT as a gait analysis system.

Related disease	Experimental models	Number of publications
Traumatic brain injury (TBI)	(a) Controlled cortical impact (b) Repeated closed-head injury (c) Single closed-head injury (d) Blast exposure injury	20
Stroke	(a) Photothrombotic (b) Ischemic (c) Chronic (d) Middle cerebral artery occlusion (MCAO) (e) Distal MCAO (f) Subarachnoid hemorrhage	11
Sciatic nerve injury (SNI)	(a) Sciatic nerve axonotmesis (b) Sciatic nerve constriction injury (c) Sciatic nerve neurotmesis (d) Sciatic nerve fibrosis	20
Spinal cord injury (SCI)	(a) Thoracic contusion (b) Thoracic hemisection (c) Thoracic laminectomy (d) Cervical hemicontusion (e) Cervical decompression (f) Cervical compression (g) TrkB.T1 astrocytic knockout	12
Parkinson’s disease (PD)	(a) 6-hydroxydopamine (6-OHDA) neurotoxin model (b) Synthetic preformed fibrils (PFF) injections (c) Methyl-4-phenyl-1,2,3,6-tetrahydropyridine (MPTP) neurotoxin model (d) R1441C LRRK2 transgenic mouse model (e) Alpha-synuclein transgenic mouse model	15
Ataxia	(a) Ataxia type 3 (b) Ataxia type 4 (c) Ataxia type 7 (d) Ataxia type 17	13

CatWalk XT parameters not reported in any publication were not included in the circular stack bar plot. The paw statistic parameters of each paw were clustered together. Print position parameters were clustered together, as were the different coupling parameters. Phase dispersion and phase lag parameters were also clustered together. In addition, parameters that were measured by different methods but had similar meanings were clustered

together: Run Duration and Other Statistics Duration; Run Average Speed, Other Statistics Average Speed, and Body Speed Mean; Run Maximum Variation, Other Statistics Maximum Variation, and Body Speed Variation Mean. Likewise, gait parameters related to non-paw contact to the walkway (the contact of hip, knee, nose, abdomen, tail, and genitalia) were clustered together. New parameters derived from the standard CatWalk XT parameters were also included. Parameters depicting asymmetric effects between the right and left limbs were clustered together. Normalized or scaled parameters (e.g., normalized by body length, size, or weight) were clustered with the equivalent non-normalized parameters. Gait parameters related to paw pixel intensity are highly influenced by the experimental settings, and therefore, they were not included in the circular stack bar plot. As a result, the total number of gait parameters presented in the circular stack bar plot is 58.

From these 58 gait parameters and all collected publications, the total number of CatWalk XT gait parameters reported for each disease is depicted in a horizontal bar plot. Depending on the direction of change, these parameters were classified as significant increase, significant decrease, and no significant difference. In the event that one publication reported a significant increase in a given gait parameter, whereas another publication reported the opposite, the visualization in the horizontal bar plot was determined based on the number of publications reporting the increase vs. decrease. Such discrepancy between studies may be due to the different experimental settings and designs and the use of animals of different strains, ages, and sex.

**FIGURE 3:**
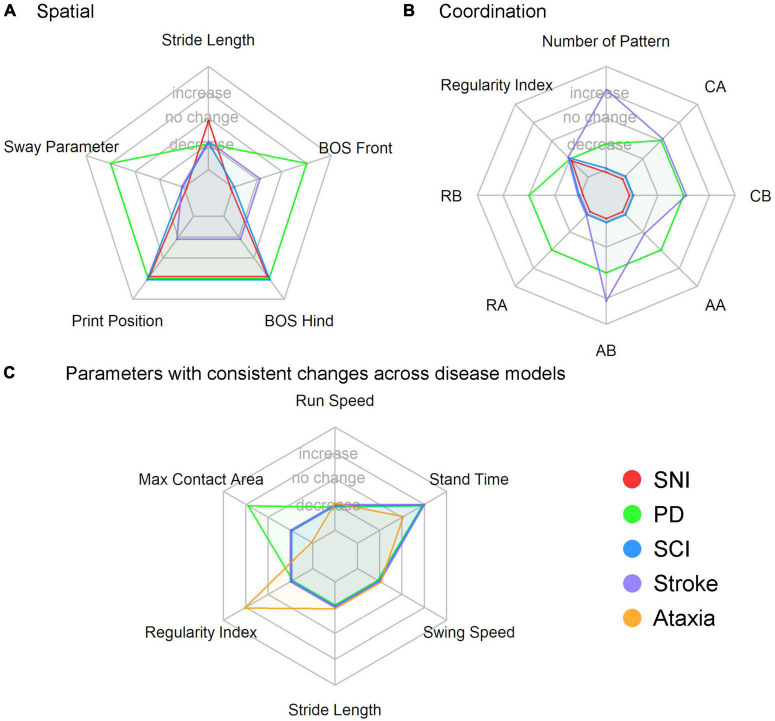
Spider plot of **(A)** spatial gait parameters (5 parameters), **(B)** coordination gait parameters (eight parameters), **(C)** six parameters which showed consistent changes within a disease model in Figure 2. BOS, base of support; CA, cruciate A; CB, cruciate B; AA, alternate A; AB, alternate B; RA, rotate A; RB, rotate B.

**FIGURE 4:**
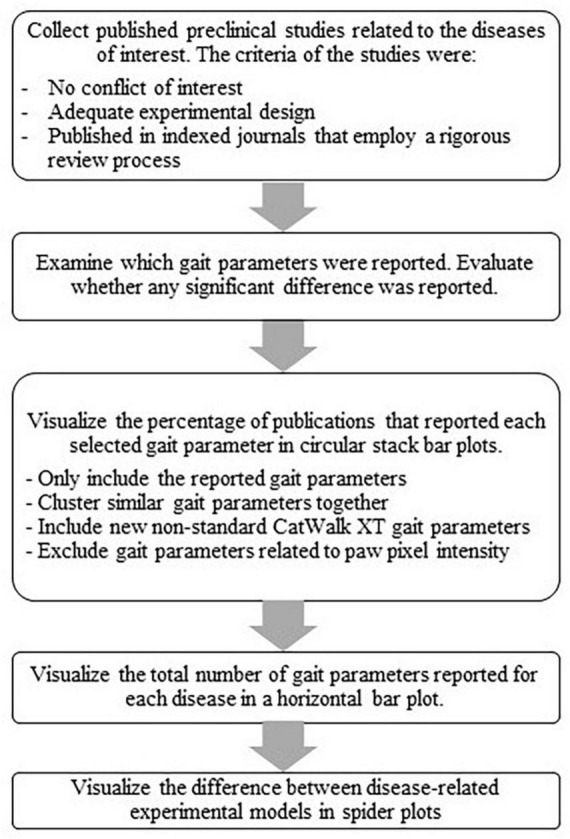
Flow chart of the review work process.

We used spider plots to visualize the differences between disease-related experimental models (Figure 3). The spider plots visualize whether each gait parameter increases, decreases, lacks significant change, or was not reported. The decision if a gait parameter was considered to increase or decrease is based on the data presented in Figure 2 (circular bar plot). Here, we show the circular plot of two parameter groups, i.e., spatial and coordination. The number of disease-related experiment models was limited to enable an understandable visualization of spider plots for novice users. Additionally, we chose several parameters based on the previous circular bar plots and visualized them in a spider plot. The review process is depicted in Figure 4.

## 3. Results

### 3.1. Reported gait parameters for each disease model

The reported CatWalk XT gait parameters were visualized using circular stack bar plots, as shown in Figure 2. The corresponding gait parameter names and groupings of parameters are given in Table 1. Following are the parameters that are reported in all six diseases.

**FIGURE 5:**
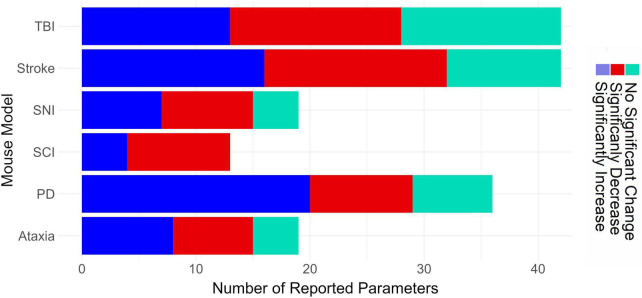
Horizontal bar graph depicting the total number of reported gait parameters from the 58 gait parameters listed in Table 1. TBI, traumatic brain injury; SNI, sciatic nerve injury; SCI, spinal cord injury; PD, Parkinson’s disease.

The total number of reported gait parameters for each disease is visualized in Figure 5. Studies of TBI and stroke reported considerably more gait parameters than any other disease model, a total of 42 gait parameters. Out of these, stroke studies reported the highest number of gait parameters showing a significant difference between injured and control groups, i.e., 32. By contrast, studies on SCI reported fewer gait parameters, i.e., 13.

### 3.2. Comparing gait parameters between disease models

Figures 3A, B show the spider plots of the spatial and coordination parameters for four different disease models. These plots inform us if a gait parameter is mostly reported as having a significant increase, significant decrease, no significant difference, or was not reported. Overall, the results show that stride length significantly decreases in PD, SCI, and stroke but not in SNI. The base of support (BOS) of the front paws increases in PD but decreases in stroke and is not reported in SNI and SCI. BOS of hind paws and print position increase in SNI, PD, and SCI but decreases in stroke. The sway parameter is a new secondary spatial parameter developed for PD (Timotius et al., 2018b); therefore, it is not reported in the other models. In the spider plot for coordination parameters, the regularity index (RI) decreases in all diseases, and RI was the only coordination parameter reported in SNI and SCI studies.

Based on the circular bar plots in Figure 2, several gait parameters show a consistent direction of change within a disease model, and these parameters are reported by at least 25% of the publications in the respective disease. For example, all PD publications that reported a significant change in swing speed reported a decreasing trend. These gait parameters include run speed (always decreased in stroke), stand time (always increased in SCI and PD), swing speed (always decreased in stroke, SCI, and PD), stride length (always decreased in stroke, SCI, and ataxia), regularity index (always decreased in SCI), maximum contact area (always decreased in stroke). We generated a separate spider plot for these parameters for stroke, SCI, PD, and ataxia (Figure 3C).

## 4. Discussion

Automated gait analysis systems, such as CatWalk XT or DigiGait™, measure hundreds of different parameters. Therefore, one of the main challenges is identifying the relevant parameters for a specific disease model. The present work was designed to facilitate an efficient literature-based selection of CatWalk XT parameters for future studies in several mouse models. To do so, this study performed a comprehensive review of reported gait abnormalities for six PNS and CNS diseases: TBI, stroke, SNI, SCI, PD, and ataxia.

Our findings suggest that certain parameters may be more appropriate than others for specific models, while others may not be as useful. For example, the average duty cycle in TBI showed limited evidence of significant change, while the average swing time in TBI showed mixed results. The average BOS of the hind paws also showed limited promise for TBI studies, with the majority of reports indicating no significant change. In contrast, the average BOS of the hind paws in ataxia and average swing time in SNI had more consistent evidence of significant change (increase), suggesting their potential usefulness in those models. Similarly, the increasing average BOS of the hind paws in SCI and decreasing average toe spread in SNI had more consistent evidence of significant change, indicating that these parameters may be more relevant for those models. The change in hind paw BOS or the absence of change in the different models (see Figure 3A) may reflect different compensatory mechanisms to restore balance, as can be seen in different models such as ataxia (Kyriakou et al., 2016) and stroke (Zampogna et al., 2020). Additionally, there were more reports of significant change (decrease or increase) in the average BOS of the front paws in stroke than no significant change, while for the same parameter in TBI and PD, the evidence was mixed.

Our review also suggests that certain gait parameters may serve as potential indicators of gait dysfunction across multiple disease models, while others may be specific to particular models. For instance, changes in phase dispersion / phase lag (which describes the temporal relationship between placement of two paws within a step cycle) and the mean of max intensity at (%, relative to stand time) were consistently reported as significant in TBI and stroke models, implying that they could potentially serve as main alerting parameters of gait dysfunction in these models. In contrast, the parameter “intermediate toe spread” was distinctive to the SNI model. However, in some cases it was decreased (Knorr et al., 2021) and in others increased (Okuwa et al., 2018), maybe due to differences in the SNI models. This phenotype highlights the potential use of toe spread as a core parameter for assessing SNI-related gait dysfunction or testing novel therapies. A further finding of our review is that the less site-specific the injury at the origin of the model (e.g., TBI, stroke), the more parameters will be significantly changed by the injury, possibly due to the extensive and variable nature of the effect on brain of the insult in these models, compared to the restricted effect that is seen when a specific nerve is damaged, as in SNI. In other words, the greater the cell loss and the less site-specific the damage, the more severe the effect on the gait.

Another interesting finding is the considerable variation in the direction of gait alteration between animal models of the same disease, a variability that can be observed even when measuring the same gait parameter. One example is print area in SCI animal models, where some publications report a significant increase but others a significant decrease or no change at all. However, an increase or decrease in a single parameter may not necessarily be indicative of gait impairment or recovery (Kyriakou et al., 2016). Instead, it may be due to factors such as the disorder model (e.g., in some TBI or stroke models, the injury may vary from animal to animal depending on the method used for injury), genotype and sex of the animals used, and various experimental factors affecting the standardization of gait recordings (Wahlsten et al., 2003; O’Leary et al., 2013; Kafkafi et al., 2017).

It should be noted that while the primary focus of this work has been gait analysis in pre-clinical mouse models of human diseases, extension to rat models is theoretically plausible. For example, comparable effects on stand time and print area following peripheral nerve injury have been previously reported in both mice and rats (Heinzel et al., 2020b). In addition, a significant decrease in swing speed, duty cycle, and paw print intensity have been observed in both mouse and rat stroke models (Encarnacion et al., 2011; Hetze et al., 2012). Moreover, recent evidence suggests that it is also possible to extend gait analysis to non-motor specific neurodegenerative diseases that are primarily characterized by cognitive deficits, like Alzheimer’s disease (Koivisto et al., 2019; Castillo-Mariqueo et al., 2021; Castillo-Mariqueo and Giménez-Llort, 2022). Thus, it is very likely that, in the future, objective measurement of gait abnormalities could aid in predicting disease onset and progression and improving quality of life also in non-motor-specific diseases.

## 5. Conclusion and a proposal/Future directions

When investigating data from CatWalk XT, results that align with the literature are easier to understand and interpret. Novel findings that have not been previously reported may represent either a novel discovery or a false positive and, as such, should be further investigated. Our work sorted the existing information from the literature for that exact purpose. Nevertheless, we acknowledge that our method might be limited in its generalizability as it is based solely on reviewing published papers. More concretely, published data carries an inherent bias since it often ignores unreported or omitted information (i.e., non-significant results). Further, the comprehensive phenotyping and understanding of different mouse models of CNS and PNS damage require expertise in numerous disease areas and collaboration between experts. It is hoped that the findings of this study, derived from a comprehensive review of CatWalk XT findings, will promote the standardization of parameter selection and guide new users in selecting parameters. Another important potential bias, which was not discussed in our paper but has significant effects in gait studies, is the details of gait recording protocols, and more importantly the report of these details in publication. Part of our limitations in this paper in providing additional information and guidance is missing information in most papers on the details of used protocols; the full information (room lighting intensity; recording parameters; number of trials per animal; reinforcer used, to name but a few) will greatly help to promote standardization between CatWalk XT users and will encourage users of other systems to report their results with similar high standards. The current literature-based parameter selection should serve as an initial step in exploring further the measured gait parameters. Our next step is to conduct an in-depth meta-analysis of raw data obtained with CatWalk XT from different laboratories that are experts in the investigated models discussed here.

## Author contributions

LB, RR, and IT: conceptualization, methodology, and data analysis. LB, RR, IT, and BR-H: investigation. IT and BR-H: data collection. IT: software and data visualization. IT, RR, BR-H, LN, SH, and LB: writing—review and editing. LB, LN, and SH: supervision. All authors contributed to the article and approved the submitted version.
