# Supplementary material for: CatWalk XT gait parameters: a review of reported parameters in pre-clinical studies of multiple central nervous system and peripheral nervous system disease models
Source: Front Behav Neurosci. 2023 Jun 7;17:1147784. doi: 10.3389/fnbeh.2023.1147784 (PMC10284348; doi:10.3389/fnbeh.2023.1147784)
Supplement: Supplementary file 1 [file Data_Sheet_1.PDF]

Supplementary Material

CatWalk XT gait parameters: A review of reported parameters in pre-clinical studies of Multiple CNS and PNS Disease Models

Ivanna K. Timotius, Reinko F. Roelofs, Bar Richmond-Hacham, Lucas PJJ Noldus, Stephan von Hörsten, Lior Bikovski*

***Correspondence:** Lior Bikovski: liorbiko@tauex.tau.ac.il

In order to show that all papers reviewed were peer-reviewed, the following table present the names of all journals that the studies were used from with their respective impact factor up to 2023.

Supplementary Table 1. The names of the journals and impact factors of the studies reviewed

No	Journal Name	Impact Factor
1	Acta Neuropathologica Communications	7.581
2	Advanced Science	14.136
3	Aging	5.955
4	ASN Neuro	3.617
5	Behavioural Brain Research	3.332
6	Biochemical And Biophysical Research Communications	3.575
7	Biomaterials	12.479
8	Biomedicines	4.757
9	BMC Neuroscience	3.264
10	BMC Research Notes	1.66
11	Brain	15.26
12	Brain Research	3.252
13	Brain Structure And Function	3.27
14	Cell Death & Disease	9.696
15	Cell Transplantation	4.139
16	Critical Care	9.097
17	eLife	8.713
18	Embo Molecular Medicine	14.005
19	Eneuro	4.081
20	Frontiers In Cellular Neuroscience	5.505
21	Frontiers In Neuroscience	4.677
22	Frontiers In Physiology	4.566
23	Genes	4.096
24	Genes, Brain and Behavior	3.708
25	Brain And Behavior	2.708
26	Human Molecular Genetics	6.15

No	Journal Name	Impact Factor
27	IEEE Transactions On Biomedical Engineering	4.538
28	Injury	2.137
29	International Journal Of Neuroscience	2.292
30	JCI Insight	8.315
31	Journal Of Alzheimer's Disease	4.472
32	Journal Of Biomedical Materials Research Part A	4.396
33	Journal Of Controlled Release	11.47
34	Journal Of Ethnopharmacology	4.36
35	Journal Of Molecular Neuroscience	3.444
36	Journal Of Neurochemistry	5.372
37	Journal Of Neuroscience Methods	2.785
38	Journal Of Neurosurgery: Spine	3.467
39	Journal Of Neurotrauma	5.269
40	Journal Of Oral Science	1.68
41	Journal Of Reconstructive Microsurgery	2.37
42	Journal Of Venomous Animals And Toxins Including Tropical Diseases	2.22
43	Journal Of Visualized Experiments	1.4
44	Medical Science Monitor	1.433
45	Molecular Neurodegeneration	18.879
46	Molecular Neurobiology	5.59
47	Molecular Pain	3.774
48	Molecular Therapy - Nucleic Acids	10.183
49	Nature Communications	17.69
50	Neural Development	3.842
51	Neural Regeneration Research	5.135
52	Neurobiology Of Disease	5.227
53	Neurobiology Of Learning And Memory	3.244
54	Neuromolecular Medicine	4.103
55	Neuropharmacology	5.251
56	Neuroreport	1.343
57	Neuroscience	3.59
58	Neuroscience Letters	3.046
59	Neurotherapeutics	6.088
60	Oncotarget	5.168
61	Plastic And Reconstructive Surgery - Global Open	1.47
62	Proceedings Of The National Academy Of Sciences	12.78
63	Science Translational Medicine	19.32
64	Scientific Reports	4.996
65	Stem Cell Reviews And Reports	6.692
66	The Journal Of Neuroscience	6.709
67	Ultrasound In Medicine & Biology	3.694

The journal impact factor and their Scopus CiteScore are shown in the following figures.

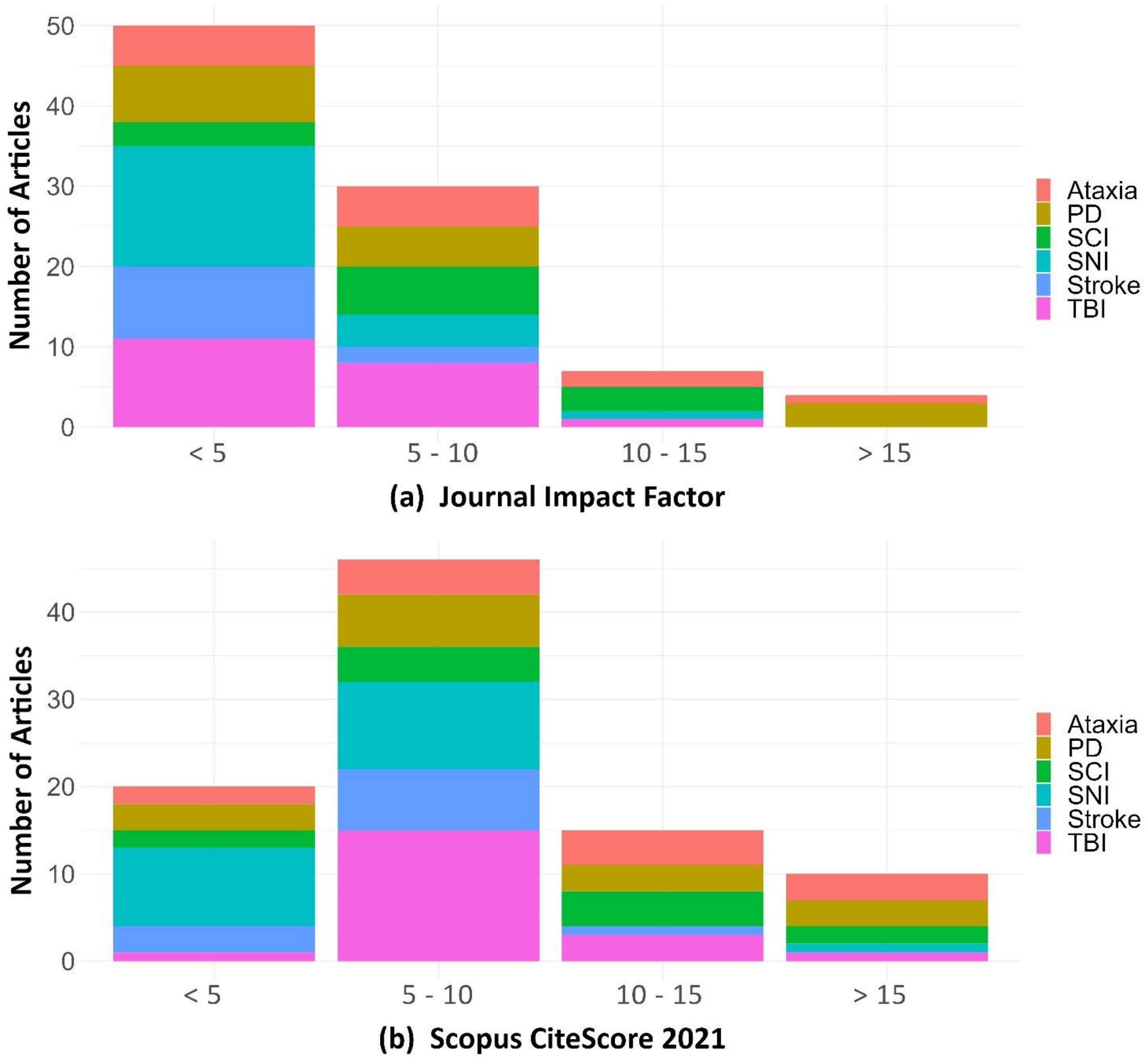

Supplementary Figure 1. Journal Impact Factor and Scopus CiteScore of the articles included in the study.

Here, we show the circular bar plots as shown in Figure 2, and the corresponding tables. The names of corresponding gait parameters are listed in Table 2 in the main article.

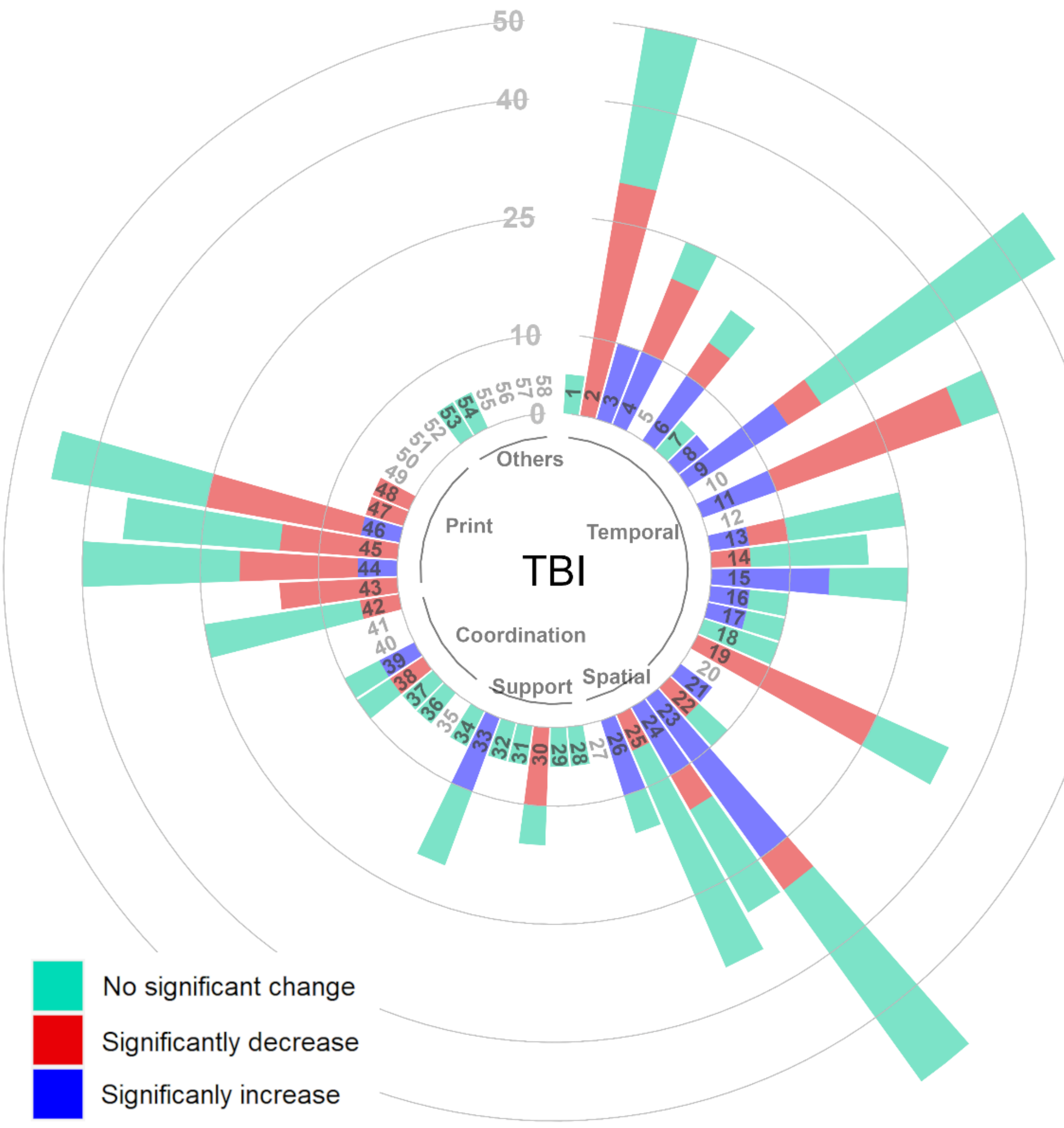

Supplementary Figure 2. Circular bar plot depicting the percentage of publications reporting specific CatWalk XT gait parameters for TBI.

Supplementary Table 2. The Number of Publications Reporting Specific CatWalk XT Gait Parameters for TBI (sig.: significant)

No	CatWalk XT Parameters	No sig. change	*Sig. increase*	*Sig. decrease*
1	Run Duration / Other Statistics Duration	1	0	0
2	Run Average Speed / Other Statistics Average Speed / Body Speed Mean	4	0	6
3	Run Max. Variation / Other Statistics Max. Variation / Body Speed Variation Mean	0	2	0
4	Stand (s) Mean	1	2	2
5	Stand (s) SD	0	0	0
6	Stand Index Mean	1	2	1
7	Max Contact At (%) Mean	1	0	0
8	Max Intensity At (%) Mean	0	1	0
9	Swing (s) Mean	7	3	1
10	Swing (s) SD	0	0	0
11	Swing Speed (cm/s) Mean	1	2	5
12	Swing Speed (cm/s) SD	0	0	0
13	Step Cycle (s) Mean	3	1	1
14	Duty Cycle (%) Mean	3	0	1
15	Single Stance (s) Mean	2	3	0
16	Initial Dual Stance (s) Mean	1	1	0
17	Terminal Dual Stance (s) Mean	1	1	0
18	Other Statistics Number of Steps	2	0	0
19	Other Statistics Cadence	2	0	5
20	RM Right Hip / RK Right Knee / LM Left Hip / LK Left Knee / NO Nose / AB Abdomen / TA Tail / GT Genitalia (%)	0	0	0
21	Phase Dispersions / Phase Lag	0	1	0
22	Couplings	1	0	1
23	Stride Length (cm) Mean or Normalized Stride Length	6	5	1
24	BOS Front Paws Mean (cm)	3	2	1
25	BOS Hind Paws Mean (cm)	6	0	1
26	Print Positions	1	2	0
27	Sway parameters	0	0	0
28	Support Zero (%)	1	0	0
29	Support Single (%)	1	0	0
30	Support Diagonal (%)	1	0	2
31	Support Girdle (%)	1	0	0
32	Support Lateral (%)	1	0	0
33	Support Three (%)	2	2	0
34	Support Four (%)	1	0	0
35	Step Sequence Number of Patterns	0	0	0
36	Step Sequence CA (%)	1	0	0
37	Step Sequence CB (%)	1	0	0
38	Step Sequence AA (%)	1	0	1
39	Step Sequence AB (%)	1	1	0
40	Step Sequence RA (%)	0	0	0
41	Step Sequence RB (%)	0	0	0
42	Step Sequence Regularity Index (%)	4	0	1
43	Max Contact Area (cm^2) Mean	0	0	3
44	Print Length (cm) Mean	4	1	3
45	Print Width (cm) Mean	4	0	3
46	Print Area (cm^2) Mean	4	1	4
47	Toe Spread (cm) Mean	0	0	1
48	Paw Angle Body Axis (°) Mean	0	0	1
49	Paw Angle Movement Vector (°) Mean	0	0	0
50	Intermediate toe spread	0	0	0
51	Sciatic functional index (SFI)	0	0	0
52	Fibular Functional Index (FFI)	0	0	0
53	Intensity ratio and asymmetry	1	0	0
54	Difference Score	1	0	0
55	Combined CatWalk Index (CCI)	0	0	0

No	CatWalk XT Parameters	No sig. change	*Sig. increase*	*Sig. decrease*
56	Stand ratio / Stride Length ratio / Step Cycle ratio / Swing Speed ratio / Print Length ratio / Print Width ratio	0	0	0
57	Phase Dispersion asymmetry / Couplings asymmetry	0	0	0
58	Print Area RH/LH Ratio or Duty Cycle RH/LH Ratio	0	0	0

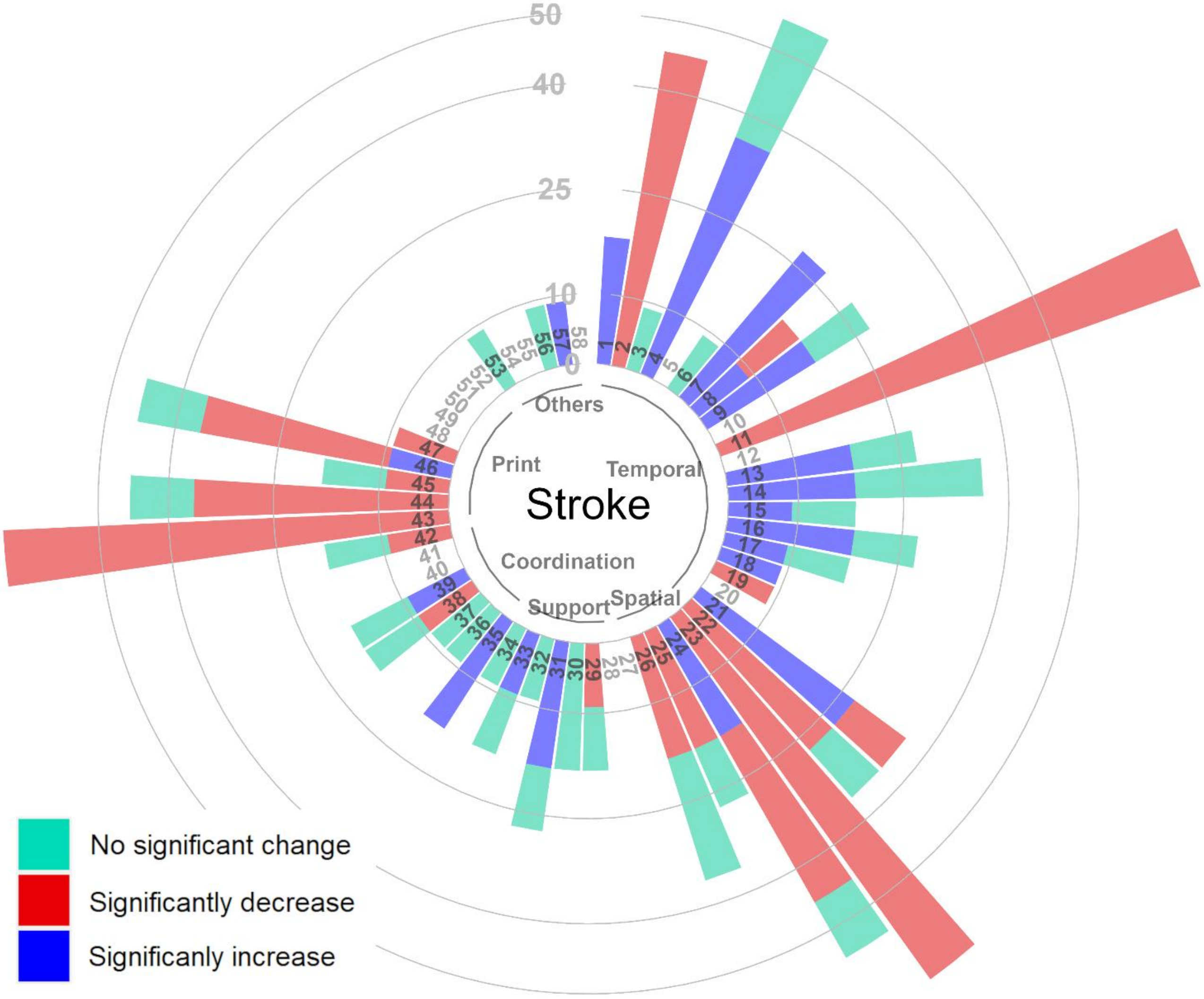

Supplementary Figure 3. Circular bar plot depicting the percentage of publications reporting specific CatWalk XT gait parameters for Stroke

Supplementary Table 3. The Number of Publications Reporting Specific CatWalk XT Gait Parameters for Stroke (sig.: significant)

No	CatWalk XT Parameters	No sig. change	*Sig. increase*	*Sig. decrease*
1	Run Duration / Other Statistics Duration	0	2	0
2	Run Average Speed / Other Statistics Average Speed / Body Speed Mean	0	0	5
3	Run Max. Variation / Other Statistics Max. Variation / Body Speed Variation Mean	1	0	0
4	Stand (s) Mean	2	4	0
5	Stand (s) SD	0	0	0
6	Stand Index Mean	1	0	0
7	Max Contact At (%) Mean	0	3	0
8	Max Intensity At (%) Mean	0	1	1
9	Swing (s) Mean	1	2	0
10	Swing (s) SD	0	0	0
11	Swing Speed (cm/s) Mean	0	0	8
12	Swing Speed (cm/s) SD	0	0	0
13	Step Cycle (s) Mean	1	2	0
14	Duty Cycle (%) Mean	2	2	0
15	Single Stance (s) Mean	1	1	0
16	Initial Dual Stance (s) Mean	1	2	0
17	Terminal Dual Stance (s) Mean	1	1	0
18	Other Statistics Number of Steps	0	1	0
19	Other Statistics Cadence	0	0	1
20	RM Right Hip / RK Right Knee / LM Left Hip / LK Left Knee / NO Nose / AB Abdomen / TA Tail / GT Genitalia (%)	0	0	0
21	Phase Dispersions / Phase Lag	0	3	1
22	Couplings	1	0	3
23	Stride Length (cm) Mean or Normalized Stride Length	0	0	7
24	BOS Front Paws Mean (cm)	1	2	3
25	BOS Hind Paws Mean (cm)	1	0	2
26	Print Positions	2	0	2
27	Sway parameters	0	0	0
28	Support Zero (%)	0	0	0
29	Support Single (%)	1	0	1
30	Support Diagonal (%)	2	0	0
31	Support Girdle (%)	1	2	0
32	Support Lateral (%)	1	0	0
33	Support Three (%)	1	1	0
34	Support Four (%)	1	0	0
35	Step Sequence Number of Patterns	0	2	0
36	Step Sequence CA (%)	1	0	0
37	Step Sequence CB (%)	1	0	0
38	Step Sequence AA (%)	1	0	1
39	Step Sequence AB (%)	1	1	0
40	Step Sequence RA (%)	0	0	0
41	Step Sequence RB (%)	0	0	0
42	Step Sequence Regularity Index (%)	1	0	1
43	Max Contact Area (cm^2) Mean	0	0	7
44	Print Length (cm) Mean	1	0	4
45	Print Width (cm) Mean	1	0	1
46	Print Area (cm^2) Mean	1	1	3
47	Toe Spread (cm) Mean	0	0	1
48	Paw Angle Body Axis (°) Mean	0	0	0
49	Paw Angle Movement Vector (°) Mean	0	0	0
50	Intermediate toe spread	0	0	0
51	Sciatic functional index (SFI)	0	0	0
52	Fibular Functional Index (FFI)	0	0	0
53	Intensity ratio and asymmetry	1	0	0
54	Difference Score	0	0	0
55	Combined CatWalk Index (CCI)	0	0	0

No	CatWalk XT Parameters	No sig. change	*Sig. increase*	*Sig. decrease*
56	Stand ratio / Stride Length ratio / Step Cycle ratio / Swing Speed ratio / Print Length ratio / Print Width ratio	1	0	0
57	Phase Dispersion asymmetry / Couplings asymmetry	0	1	0
58	Print Area RH/LH Ratio or Duty Cycle RH/LH Ratio	0	0	0

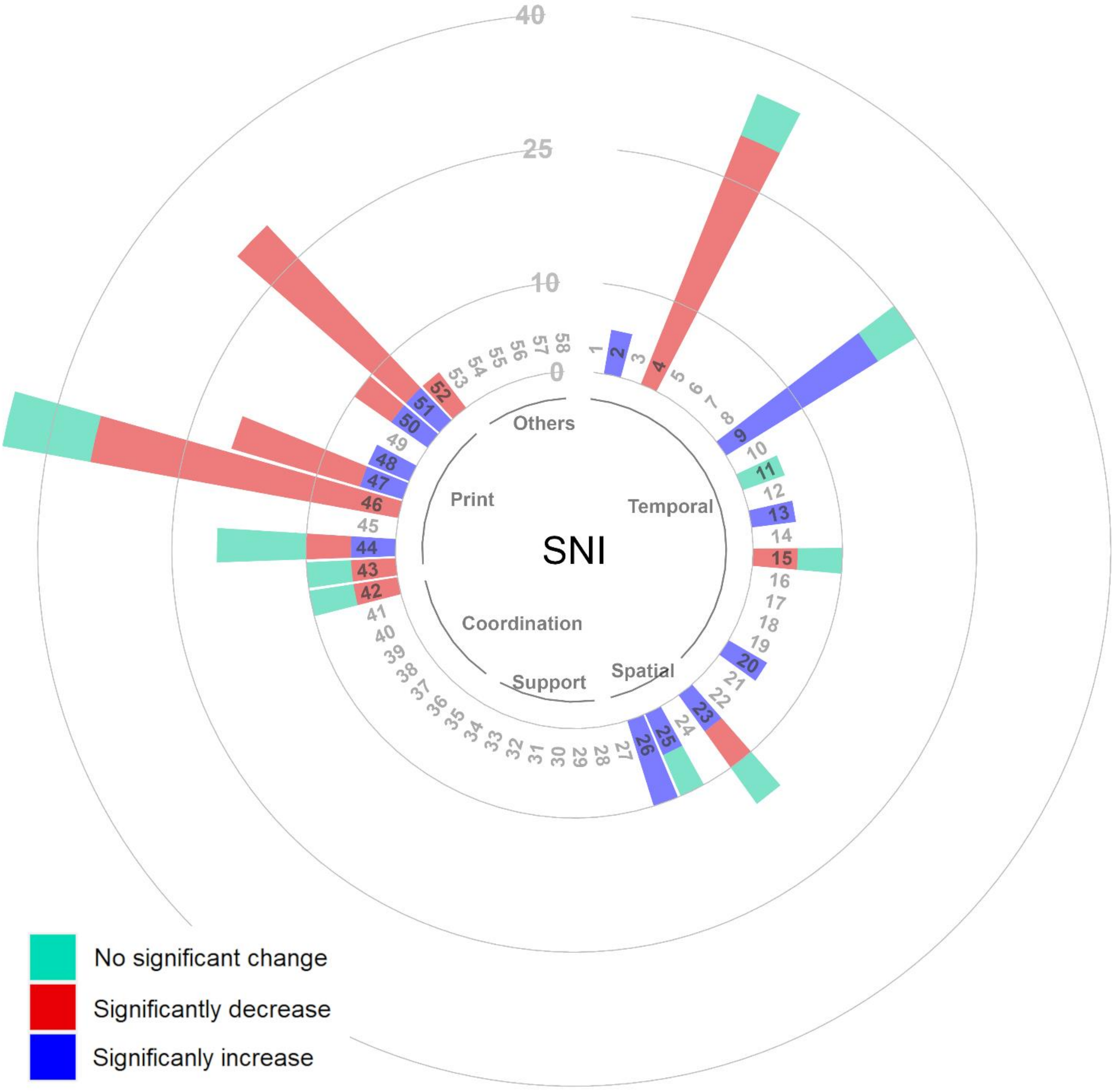

Supplementary Figure 4. Circular bar plot depicting the percentage of publications reporting specific CatWalk XT gait parameters for SNI

Supplementary Table 4. The Number of Publications Reporting Specific CatWalk XT Gait Parameters for SNI (sig.: significant)

No	CatWalk XT Parameters	No sig. change	*Sig. increase*	*Sig. decrease*
1	Run Duration / Other Statistics Duration	0	0	0
2	Run Average Speed / Other Statistics Average Speed / Body Speed Mean	0	1	0
3	Run Max. Variation / Other Statistics Max. Variation / Body Speed Variation Mean	0	0	0
4	Stand (s) Mean	1	0	6
5	Stand (s) SD	0	0	0
6	Stand Index Mean	0	0	0
7	Max Contact At (%) Mean	0	0	0
8	Max Intensity At (%) Mean	0	0	0
9	Swing (s) Mean	1	4	0
10	Swing (s) SD	0	0	0
11	Swing Speed (cm/s) Mean	1	0	0
12	Swing Speed (cm/s) SD	0	0	0
13	Step Cycle (s) Mean	0	1	0
14	Duty Cycle (%) Mean	0	0	0
15	Single Stance (s) Mean	1	0	1
16	Initial Dual Stance (s) Mean	0	0	0
17	Terminal Dual Stance (s) Mean	0	0	0
18	Other Statistics Number of Steps	0	0	0
19	Other Statistics Cadence	0	0	0
20	RM Right Hip / RK Right Knee / LM Left Hip / LK Left Knee / NO Nose / AB Abdomen / TA Tail / GT Genitalia (%)	0	1	0
21	Phase Dispersions / Phase Lag	0	0	0
22	Couplings	0	0	0
23	Stride Length (cm) Mean or Normalized Stride Length	1	1	1
24	BOS Front Paws Mean (cm)	0	0	0
25	BOS Hind Paws Mean (cm)	1	1	0
26	Print Positions	0	2	0
27	Sway parameters	0	0	0
28	Support Zero (%)	0	0	0
29	Support Single (%)	0	0	0
30	Support Diagonal (%)	0	0	0
31	Support Girdle (%)	0	0	0
32	Support Lateral (%)	0	0	0
33	Support Three (%)	0	0	0
34	Support Four (%)	0	0	0
35	Step Sequence Number of Patterns	0	0	0
36	Step Sequence CA (%)	0	0	0
37	Step Sequence CB (%)	0	0	0
38	Step Sequence AA (%)	0	0	0
39	Step Sequence AB (%)	0	0	0
40	Step Sequence RA (%)	0	0	0
41	Step Sequence RB (%)	0	0	0
42	Step Sequence Regularity Index (%)	1	0	1
43	Max Contact Area (cm²) Mean	1	0	1
44	Print Length (cm) Mean	2	1	1
45	Print Width (cm) Mean	0	0	0
46	Print Area (cm²) Mean	2	0	7
47	Toe Spread (cm) Mean	0	1	3
48	Paw Angle Body Axis (°) Mean	0	1	0
49	Paw Angle Movement Vector (°) Mean	0	0	0
50	Intermediate toe spread	0	1	1
51	Sciatic functional index (SFI)	0	1	5
52	Fibular Functional Index (FFI)	0	0	1
53	Intensity ratio and asymmetry	0	0	0
54	Difference Score	0	0	0
55	Combined CatWalk Index (CCI)	0	0	0

No	CatWalk XT Parameters	No sig. change	*Sig. increase*	*Sig. decrease*
56	Stand ratio / Stride Length ratio / Step Cycle ratio / Swing Speed ratio / Print Length ratio / Print Width ratio	0	0	0
57	Phase Dispersion asymmetry / Couplings asymmetry	0	0	0
58	Print Area RH/LH Ratio or Duty Cycle RH/LH Ratio	0	0	0

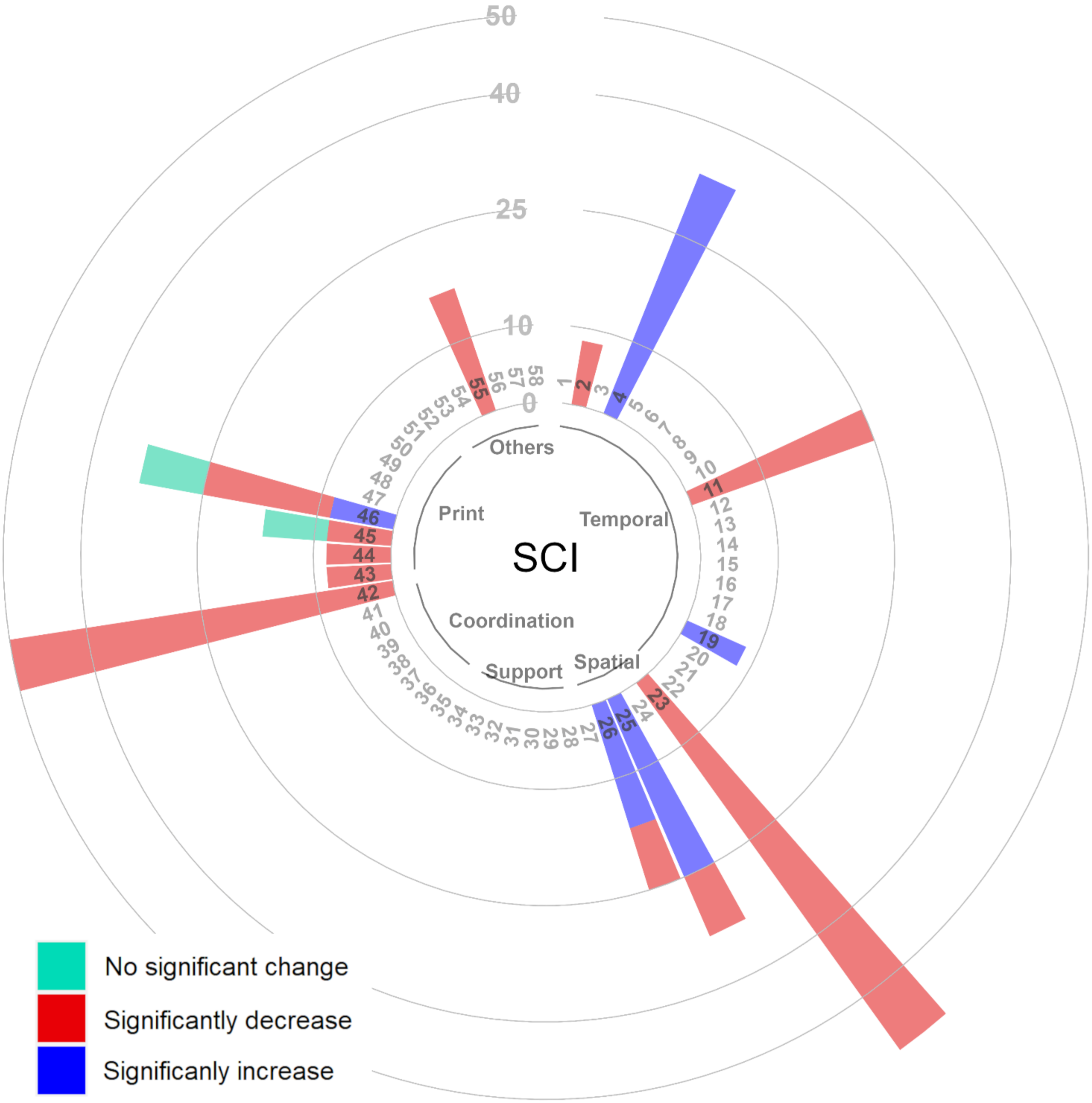

Supplementary Figure 5. Circular bar plot depicting the percentage of publications reporting specific CatWalk XT gait parameters for SCI

Supplementary Table 5. The Number of Publications Reporting Specific CatWalk XT Gait Parameters for SCI (sig.: significant)

No	CatWalk XT Parameters	No sig. change	*Sig. increase*	*Sig. decrease*
1	Run Duration / Other Statistics Duration	0	0	0
2	Run Average Speed / Other Statistics Average Speed / Body Speed Mean	0	0	1
3	Run Max. Variation / Other Statistics Max. Variation / Body Speed Variation Mean	0	0	0
4	Stand (s) Mean	0	4	0
5	Stand (s) SD	0	0	0
6	Stand Index Mean	0	0	0
7	Max Contact At (%) Mean	0	0	0
8	Max Intensity At (%) Mean	0	0	0
9	Swing (s) Mean	0	0	0
10	Swing (s) SD	0	0	0
11	Swing Speed (cm/s) Mean	0	0	3
12	Swing Speed (cm/s) SD	0	0	0
13	Step Cycle (s) Mean	0	0	0
14	Duty Cycle (%) Mean	0	0	0
15	Single Stance (s) Mean	0	0	0
16	Initial Dual Stance (s) Mean	0	0	0
17	Terminal Dual Stance (s) Mean	0	0	0
18	Other Statistics Number of Steps	0	0	0
19	Other Statistics Cadence	0	1	0
20	RM Right Hip / RK Right Knee / LM Left Hip / LK Left Knee / NO Nose / AB Abdomen / TA Tail / GT Genitalia (%)	0	0	0
21	Phase Dispersions / Phase Lag	0	0	0
22	Couplings	0	0	0
23	Stride Length (cm) Mean or Normalized Stride Length	0	0	7
24	BOS Front Paws Mean (cm)	0	0	0
25	BOS Hind Paws Mean (cm)	0	3	1
26	Print Positions	0	2	1
27	Sway parameters	0	0	0
28	Support Zero (%)	0	0	0
29	Support Single (%)	0	0	0
30	Support Diagonal (%)	0	0	0
31	Support Girdle (%)	0	0	0
32	Support Lateral (%)	0	0	0
33	Support Three (%)	0	0	0
34	Support Four (%)	0	0	0
35	Step Sequence Number of Patterns	0	0	0
36	Step Sequence CA (%)	0	0	0
37	Step Sequence CB (%)	0	0	0
38	Step Sequence AA (%)	0	0	0
39	Step Sequence AB (%)	0	0	0
40	Step Sequence RA (%)	0	0	0
41	Step Sequence RB (%)	0	0	0
42	Step Sequence Regularity Index (%)	0	0	6
43	Max Contact Area (cm^2) Mean	0	0	1
44	Print Length (cm) Mean	0	0	1
45	Print Width (cm) Mean	1	0	1
46	Print Area (cm^2) Mean	1	1	2
47	Toe Spread (cm) Mean	0	0	0
48	Paw Angle Body Axis (°) Mean	0	0	0
49	Paw Angle Movement Vector (°) Mean	0	0	0
50	Intermediate toe spread	0	0	0
51	Sciatic functional index (SFI)	0	0	0
52	Fibular Functional Index (FFI)	0	0	0
53	Intensity ratio and asymmetry	0	0	0
54	Difference Score	0	0	0
55	Combined CatWalk Index (CCI)	0	0	2

No	CatWalk XT Parameters	No sig. change	*Sig. increase*	*Sig. decrease*
56	Stand ratio / Stride Length ratio / Step Cycle ratio / Swing Speed ratio / Print Length ratio / Print Width ratio	0	0	0
57	Phase Dispersion asymmetry / Couplings asymmetry	0	0	0
58	Print Area RH/LH Ratio or Duty Cycle RH/LH Ratio	0	0	0

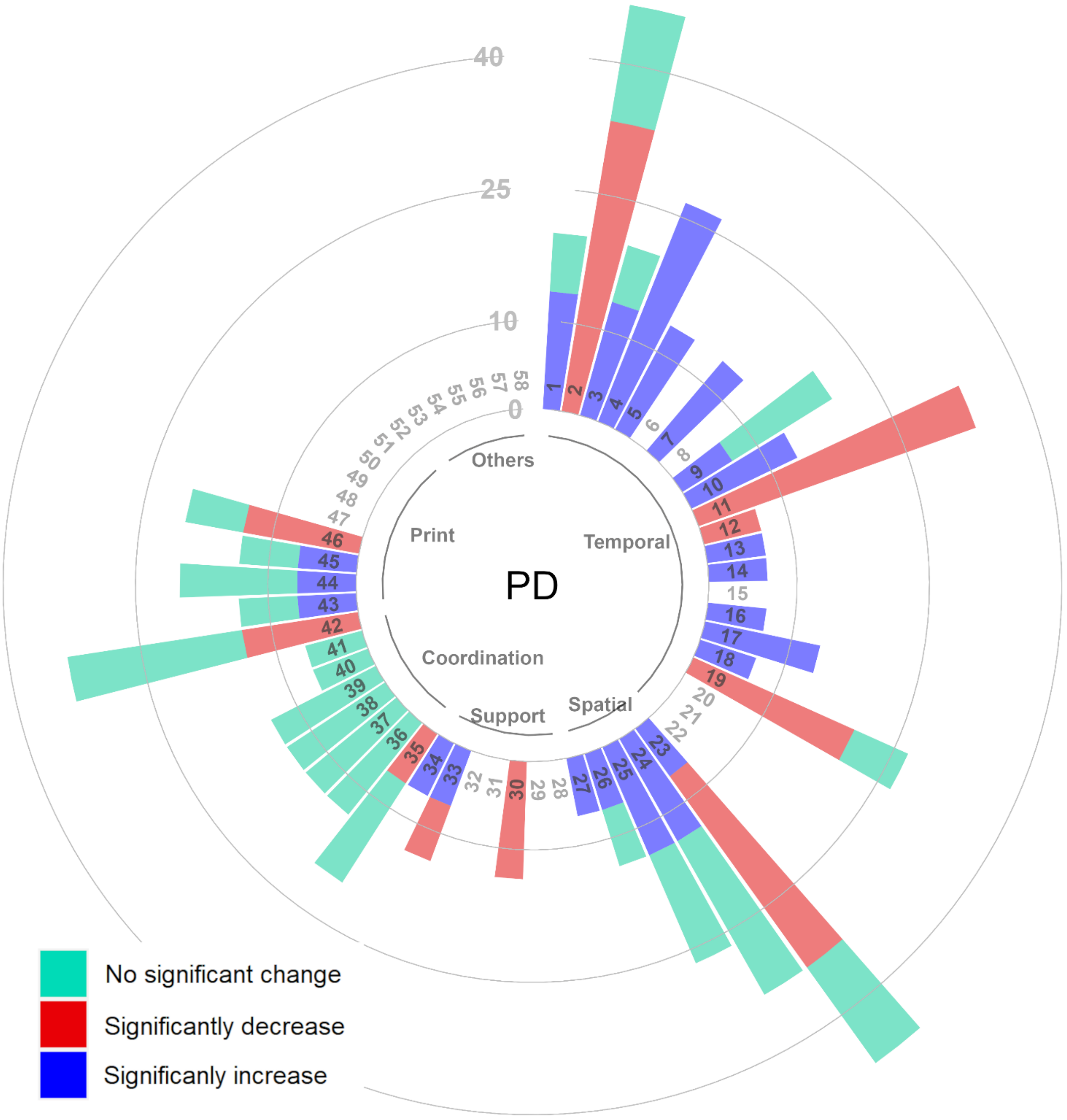

Supplementary Figure 6. Circular bar plot depicting the percentage of publications reporting specific CatWalk XT gait parameters for PD

Supplementary Table 6. The Number of Publications Reporting Specific CatWalk XT Gait Parameters for PD (sig.: significant)

No	CatWalk XT Parameters	No sig. change	*Sig. increase*	*Sig. decrease*
1	Run Duration / Other Statistics Duration	1	2	0
2	Run Average Speed / Other Statistics Average Speed / Body Speed Mean	2	0	5
3	Run Max. Variation / Other Statistics Max. Variation / Body Speed Variation Mean	1	2	0
4	Stand (s) Mean	0	4	0
5	Stand (s) SD	0	2	0
6	Stand Index Mean	0	0	0
7	Max Contact At (%) Mean	0	2	0
8	Max Intensity At (%) Mean	0	0	0
9	Swing (s) Mean	2	1	0
10	Swing (s) SD	0	2	0
11	Swing Speed (cm/s) Mean	0	0	5
12	Swing Speed (cm/s) SD	0	0	1
13	Step Cycle (s) Mean	0	1	0
14	Duty Cycle (%) Mean	0	1	0
15	Single Stance (s) Mean	0	0	0
16	Initial Dual Stance (s) Mean	0	1	0
17	Terminal Dual Stance (s) Mean	0	2	0
18	Other Statistics Number of Steps	0	1	0
19	Other Statistics Cadence	1	0	3
20	RM Right Hip / RK Right Knee / LM Left Hip / LK Left Knee / NO Nose / AB Abdomen / TA Tail / GT Genitalia (%)	0	0	0
21	Phase Dispersions / Phase Lag	0	0	0
22	Couplings	0	0	0
23	Stride Length (cm) Mean or Normalized Stride Length	2	1	4
24	BOS Front Paws Mean (cm)	3	2	0
25	BOS Hind Paws Mean (cm)	2	2	0
26	Print Positions	1	1	0
27	Sway parameters	0	1	0
28	Support Zero (%)	0	0	0
29	Support Single (%)	0	0	0
30	Support Diagonal (%)	0	0	2
31	Support Girdle (%)	0	0	0
32	Support Lateral (%)	0	0	0
33	Support Three (%)	0	1	1
34	Support Four (%)	0	1	0
35	Step Sequence Number of Patterns	2	0	1
36	Step Sequence CA (%)	2	0	0
37	Step Sequence CB (%)	2	0	0
38	Step Sequence AA (%)	2	0	0
39	Step Sequence AB (%)	2	0	0
40	Step Sequence RA (%)	1	0	0
41	Step Sequence RB (%)	1	0	0
42	Step Sequence Regularity Index (%)	3	0	2
43	Max Contact Area (cm^2) Mean	1	1	0
44	Print Length (cm) Mean	2	1	0
45	Print Width (cm) Mean	1	1	0
46	Print Area (cm^2) Mean	1	0	2
47	Toe Spread (cm) Mean	0	0	0
48	Paw Angle Body Axis (°) Mean	0	0	0
49	Paw Angle Movement Vector (°) Mean	0	0	0
50	Intermediate toe spread	0	0	0
51	Sciatic functional index (SFI)	0	0	0
52	Fibular Functional Index (FFI)	0	0	0
53	Intensity ratio and asymmetry	0	0	0
54	Difference Score	0	0	0
55	Combined CatWalk Index (CCI)	0	0	0

No	CatWalk XT Parameters	No sig. change	*Sig. increase*	*Sig. decrease*
56	Stand ratio / Stride Length ratio / Step Cycle ratio / Swing Speed ratio / Print Length ratio / Print Width ratio	0	0	0
57	Phase Dispersion asymmetry / Couplings asymmetry	0	0	0
58	Print Area RH/LH Ratio or Duty Cycle RH/LH Ratio	0	0	0

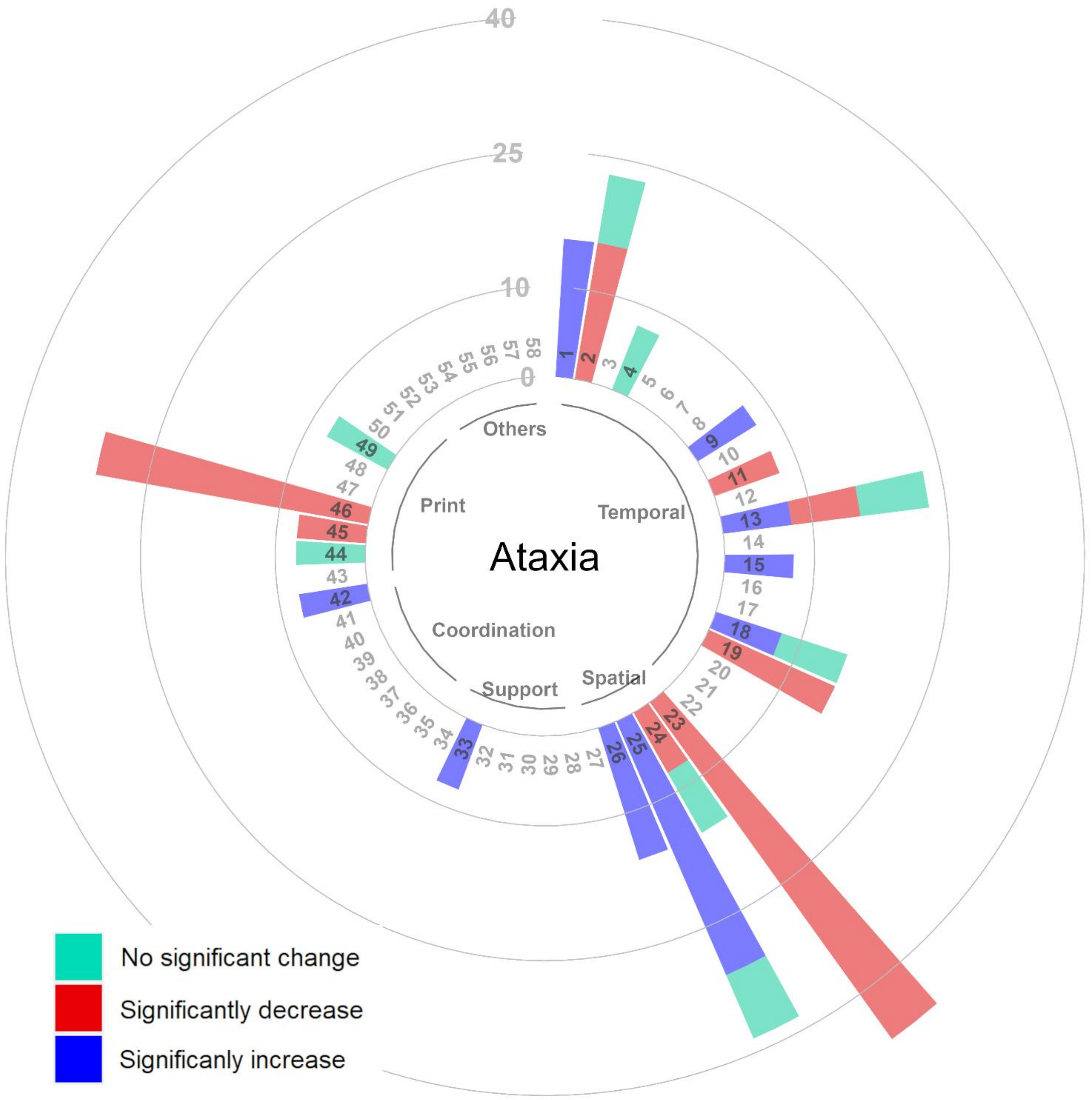

Supplementary Figure 7. Circular bar plot depicting the percentage of publications reporting specific CatWalk XT gait parameters for Ataxia

Supplementary Table 7. The Number of Publications Reporting Specific CatWalk XT Gait Parameters for Ataxia (sig.: significant)

No	CatWalk XT Parameters	No sig. change	*Sig. increase*	*Sig. decrease*
1	Run Duration / Other Statistics Duration	0	2	0
2	Run Average Speed / Other Statistics Average Speed / Body Speed Mean	1	0	2
3	Run Max. Variation / Other Statistics Max. Variation / Body Speed Variation Mean	0	0	0
4	Stand (s) Mean	1	0	0
5	Stand (s) SD	0	0	0
6	Stand Index Mean	0	0	0
7	Max Contact At (%) Mean	0	0	0
8	Max Intensity At (%) Mean	0	0	0
9	Swing (s) Mean	0	1	0
10	Swing (s) SD	0	0	0
11	Swing Speed (cm/s) Mean	0	0	1
12	Swing Speed (cm/s) SD	0	0	0
13	Step Cycle (s) Mean	1	1	1
14	Duty Cycle (%) Mean	0	0	0
15	Single Stance (s) Mean	0	1	0
16	Initial Dual Stance (s) Mean	0	0	0
17	Terminal Dual Stance (s) Mean	0	0	0
18	Other Statistics Number of Steps	1	1	0
19	Other Statistics Cadence	0	0	2
20	RM Right Hip / RK Right Knee / LM Left Hip / LK Left Knee / NO Nose / AB Abdomen / TA Tail / GT Genitalia (%)	0	0	0
21	Phase Dispersions / Phase Lag	0	0	0
22	Couplings	0	0	0
23	Stride Length (cm) Mean or Normalized Stride Length	0	0	6
24	BOS Front Paws Mean (cm)	1	0	1
25	BOS Hind Paws Mean (cm)	1	4	0
26	Print Positions	0	2	0
27	Sway parameters	0	0	0
28	Support Zero (%)	0	0	0
29	Support Single (%)	0	0	0
30	Support Diagonal (%)	0	0	0
31	Support Girdle (%)	0	0	0
32	Support Lateral (%)	0	0	0
33	Support Three (%)	0	1	0
34	Support Four (%)	0	0	0
35	Step Sequence Number of Patterns	0	0	0
36	Step Sequence CA (%)	0	0	0
37	Step Sequence CB (%)	0	0	0
38	Step Sequence AA (%)	0	0	0
39	Step Sequence AB (%)	0	0	0
40	Step Sequence RA (%)	0	0	0
41	Step Sequence RB (%)	0	0	0
42	Step Sequence Regularity Index (%)	0	1	0
43	Max Contact Area (cm^2) Mean	0	0	0
44	Print Length (cm) Mean	1	0	0
45	Print Width (cm) Mean	0	0	1
46	Print Area (cm^2) Mean	0	0	4
47	Toe Spread (cm) Mean	0	0	0
48	Paw Angle Body Axis (°) Mean	0	0	0
49	Paw Angle Movement Vector (°) Mean	1	0	0
50	Intermediate toe spread	0	0	0
51	Sciatic functional index (SFI)	0	0	0
52	Fibular Functional Index (FFI)	0	0	0
53	Intensity ratio and asymmetry	0	0	0
54	Difference Score	0	0	0
55	Combined CatWalk Index (CCI)	0	0	0

No	CatWalk XT Parameters	No sig. change	*Sig. increase*	*Sig. decrease*
56	Stand ratio / Stride Length ratio / Step Cycle ratio / Swing Speed ratio / Print Length ratio / Print Width ratio	0	0	0
57	Phase Dispersion asymmetry / Couplings asymmetry	0	0	0
58	Print Area RH/LH Ratio or Duty Cycle RH/LH Ratio	0	0	0
